# Autonomy Challenges in Epigenetic Risk-Stratified Cancer Screening: How Can Patient Decision Aids Support Informed Consent?

**DOI:** 10.3390/jpm9010014

**Published:** 2019-02-18

**Authors:** Maaike Alblas, Maartje Schermer, Yvonne Vergouwe, Ineke Bolt

**Affiliations:** 1 Department of Public Health, Erasmus MC, P.O. 2040, 3000 CA Rotterdam, The Netherlands; y.vergouwe@erasmusmc.nl; 2 Department of Medical Ethics and Philosophy of Medicine, Erasmus MC, P.O. 2040, 3000 CA Rotterdam, The Netherlands; m.schermer@erasmusmc.nl (M.S.); l.bolt@erasmusmc.nl (I.B.)

**Keywords:** cancer screening programmes, epigenetics, risk prediction, patient autonomy, informed consent, patient decision aids

## Abstract

Information of an individual’s epigenome can be useful in cancer screening to enable personalised decision making on participation, treatment options and further screening strategies. However, adding this information might result in complex risk predictions on multiple diseases, unsolicited findings and information on (past) environmental exposure and behaviour. This complicates informed consent procedures and may impede autonomous decision-making. In this article we investigate and identify the specific features of epigenetic risk-stratified cancer screening that challenge the current informed consent doctrine. Subsequently we describe current and new informed consent models and the principle of respect for autonomy and argue for a specific informed consent model for epigenetic risk-stratified screening programmes. Next, we propose a framework that guides the development of Patient Decision Aids (PDAs) to support informed consent and promote autonomous choices in the specific context of epigenetic cancer screening programmes.

## 1. Introduction

Screening programmes are important means in the prevention and early detection of several high-risk cancers [1,2,3]. Over the past few decades, there has been a transition from screening for a single cancer-type based on available patient characteristics, to screening for multiple cancers based on epigenetic testing [4,5]. Adding information from the epigenome to population-based cancer screening programmes can eventually lead to more personalised risk predictions and treatment advice [6]. Although these new screening programmes may improve the early detection and prevention of cancer, there is a drawback regarding informed consent. The screening programme will include risk assessments, and therefore also difficult risk predictions that are hard to interpret for a lay person, but can also include unsolicited findings (i.e., findings that are discovered unintentionally and may have medical, psychological and social consequences) and may reveal information about environmental exposures and behaviour from the past [7]. These features challenge existing informed consent procedures and call for ways to restructure and enrich informed consent procedures, with the ultimate goal that informed consent represents an autonomous choice.

Patient Decision Aids (PDAs) can support autonomous decision making [8,9,10]. They are proven to be effective in terms of improving knowledge and increasing patient participation [8,9,11]. However, improving knowledge and understanding does not automatically lead to autonomous choices, since an autonomous choice should be an intentional, voluntary choice that is made with sufficient understanding. Additionally, the choice should be consistent with one’s values. Vos et al. discussed that the design of current PDAs may not support autonomous decision making because (1) they often utilise explicit value clarification methods (VCM), which may lead to constructed preferences that are not congruent with one’s actual values, and (2) they mainly focus on deliberative processes instead of combining deliberation and intuition [12]. These findings have implications for PDAs in general, but these implications may be even more substantial for PDAs in epigenetic cancer screening given the additional challenges that are posed on informed consent in this context. Therefore, the aim of this study is to create a framework that guides the development of PDAs to support informed consent and promote autonomous choices when people face epigenetic risk-tailored cancer screening decisions regarding participating in such tests. 

First, we identify specific elements of epigenetic risk-stratified cancer screening that complicate informed consent procedures. Next, we provide a brief overview of the literature on existing decision aids, informed consent models and the principle of respect for autonomy, specifically in the context of cancer screening programmes and argue for a specific informed consent model suitable for the context of epigenetic cancer screening programmes. Subsequently, we identify different processes in PDAs to evaluate which ones sustain autonomous choices and the informed consent process in this context of epigenetic cancer screening. Finally, we develop a framework that can serve as starting point for designing PDAs to be used within the process of obtaining informed consent, in the specific context of new epigenetic risk-stratified cancer screening programmes.

## 2. Epigenetic Risk-Stratified Cancer Screening

New studies are currently exploring the possibility of using epigenetic markers for risk-stratification in nationwide screening programmes [5,7]. The most commonly studied epigenetic changes are DNA methylation, the addition of a methyl group to specific regions of the genome. These changes affect gene expression without changing the underlying DNA sequence. Epigenetic changes are implicated in tumour development and its progression [7,13]. Unlike genetic mutations such as *BRCA1/BRCA2* that are not reversible, changes in epigenetic markers are reversible and do not cause alterations in the underlying DNA sequence [14]. For instance in breast cancer, a women can inherit a *BRCA1/BRCA2* mutation, which gives her a life-time elevated risk of breast cancer of 50–85%[15]. Besides preventive surgery, she cannot lower this risk by herself and the mutation will always be present in her DNA sequence. This is different in epigenetic markers, that can be influenced by heritable factors and non-heritable factors such as lifestyle, and can therefore change during a person’s life [14]. If a specific epigenetic marker is responsible for an increased risk of breast cancer, this marker may be restored to its normal state with lifestyle interventions, hence lowering the risk of cancer [16].

There are studies looking into using epigenetic changes for risk assessment in risk-stratified screening programmes. Risk-stratified screening means that eligibility, frequency and modality are tailored according to one’s risk level [5]. Using epigenetic markers in risk stratified screening creates opportunities to incorporate advice on specific preventative actions [16]. Epigenetic markers hold valuable information on an individuals’ exposure to e.g., obesity, smoking, and the use of exogenous hormones. This information from the epigenome can serve as a surrogate for more subjective data on lifestyle exposure such as questionnaire data that is prone to recall bias, and might improve the performance of cancer risk prediction models as such epigenetic markers will give more accurate risk prediction than risk assessment based on questionnaire based informed risk factors [5,7].

When epigenetic information is incorporated in nationwide screening programmes, the results of these screening programmes will differ from the programmes that we are currently familiar with, such as breast cancer screening based on mammography. We want to illustrate this with a screening test which is under development, the FORECEE Women’s cancer risk Identification (WID) test [17]. In short, the WID test aims to predict the risk of developing breast, ovarian, endometrial and cervical cancer by using (epi)genetic information that is obtained with a pap smear [17]. The results of this test will include risk predictions on the four cancers, but may also include unsolicited findings (e.g., the risk of lung cancer). The debate regarding returning these unsolicited findings is still ongoing, and there is currently no consensus on the most appropriate disclosure policy [18]

Based on this description of epigenetic risk-stratified cancer screening programmes, we can discern the following features of these type of screening tests. (I) The results of the test will not only consist of a diagnosis, but will also contain risk predictions on multiple (malignant) diseases. These risk predictions pose additional challenges on patients’ ability to make autonomous choices. Several studies have shown that people are more fallible decision makers than was often assumed. They incorporate emotions and perceptions into their decision making process and, for instance, label certain numbers with affective cues such as high versus low, especially if the decision involves risk predictions [12,19]. (II) The results of the test will provide insights in specific lifestyle factors that might have contributed to an elevated risk. These insights could provoke feelings of guilt, regret or shame. These feelings might be even more substantial if one’s lifestyle has caused a so-called epigenetic inheritance (i.e., one’s lifestyle has caused a high risk of a certain type of cancer among its offspring). (III) The resulting risk score might be lowered by lifestyle because of the reversible nature of DNA methylation. However, changing someone’s lifestyle is not as simple as it seems and people might need executive capacities to set such change in motion [20]. These capacities are likely to vary between people with a low versus high social economic position, which might lead to increasing levels of inequity [21]. (IV) The results of the test might contain unsolicited findings with large variability in severity and consequences. Although unsolicited findings are also common in other tests, the specific combination of unsolicited findings, multiple diseases and risk predictions in epigenetic cancer screening leads to an increased level of complexity.

Although these elements facilitate more personalised nationwide screening programmes, they also endanger informed consent procedures. This adds to the existing problems with informed consent in tests with genetic information and testing multiple diseases simultaneously, and in current population screening programmes [22]. Therefore, informed consent procedures should be reconsidered and supported by suitable tools that aid patients in making an autonomous choice about participation in epigenetic cancer screening programmes.

## 3. Autonomy and Informed Consent

Informed consent is an ethical and legal requirement in different areas of health care, such as clinical practice and scientific research [23]. In its legal form, informed consent is an authorisation of a certain medical intervention [23,24]. However, this form of informed consent might not align with its original goal to protect patients against harm and to ensure the autonomy of patients [23]. An ‘autonomous choice’ was described by Faden and Beauchamp as an intentional, voluntary choice that is made with understanding [23]. Informed consent is considered to be an “autonomous authorisation” when a patient intentionally, voluntarily and with sufficient understanding authorises a doctor to act. Alternative conceptions of autonomy include ‘authenticity’ as a necessary condition for an autonomous choice; a person should be able to identify herself with a choice and this choice should be in line with her personal values [25,26]. If this is not the case, the choice she makes might not represent an autonomous choice. We agree that alignment with one’s values is an important condition, in particular in the context of epigenetic screening because preferences regarding participation might vary and participation might have substantial consequences (e.g., choices regarding preventive and treatment options e.g., mastectomy, procreation, or career). Taken together, informed consent represents an autonomous choice if the underlying choice was made intentionally, voluntarily, with sufficient understanding, and is aligned with one’s personal values [23,24]. 

If we would apply these conditions of an autonomous choice to informed consent in epigenetic cancer screening programmes, the patient should at least be informed about and understand the risks, benefits, harms associated with the test, and alternative screening options before consenting to the particular medical activity [23,24]. However, this would result in an overwhelming amount of information given the before mentioned specific elements of epigenetic cancer screening (e.g., complex information on different risk predictions, unsolicited findings, and interplay of genes). Previous studies have shown that such large amounts of information could lead to ineffective informed consent procedures and might cause anxiety and confusion amongst patients [27,28]. In addition, people are likely to make mistakes, e.g., in assessing numbers on risk and benefits of medical interventions because of beliefs on the likelihood of a certain option and previous experiences with risk estimates [19,29]. A recent study showed that the majority of women tend to overestimate their baseline female cancer risk and have limited knowledge on the benefits and harms of screening with mammography [30]. This indicates that this problem is also present in current screening programmes, but we argue that it will become even more substantial in epigenetic cancer screening programmes since these programs will provide more complicated risk estimates. 

We argue for an informed consent model that is suitable to the context of epigenetic risk-stratified cancer screening. Bunnik et al. describe a tiered–layered–staged model, which was originally developed for commercial personal genome testing [31,32]. In short, the tiered–layered–staged model consists of three components. The first component is tiered, which means that people can consent to different parts of the treatment or screening, depending on their preferences. For instance, in the context of personal genome testing this could mean that patients only consent to test for diseases for which medical treatment/prevention exist (that are medically actionable), or choose to also acquire results that are not directly life-saving or medically actionable (e.g., results related to reproductive health or diseases for which no treatment is available). The second component is a layer, because the model consists of several layers of information, each layer having its own level of complexity. These layers always include a baseline layer that contains information that is necessary to make a decision with knowledge, e.g., all basic information regarding the test, including resulting risk prediction, the expected false positive/negative results, over-diagnosis and the handling of unsolicited findings. The additional layers contain more detailed and complex information and can be made available depending on the patients’ preferences. The last component is staged, which means that the informed consent contains several stages in time in which information is given. The actual informed consent could contain a certain timeframe in which patients can think about their decision. Although there is currently no empirical data on the use of the tiered–layered–staged informed consent in practice, tiered informed consent is already considered best practice in biobanking in genomics- and proteomics-based research [33,34].

We suggest using the tiered–layered–staged informed consent in the context of new epigenetic risk-stratified cancer screening to overcome the challenges that are posed to traditional informed consent. The tiered–layered–staged model overcomes these problems since it organises the information in stages and categories. However, this model mainly focusses on distributing information, instead of supporting patients in processing all provided information and relating it to their values. This processing of information is necessary because solely supplying patients with information insufficiently promotes a choice that is made with understanding and in line with their values. Next to intentionality, voluntariness and understanding, authenticity is seen as a necessary condition of the principle of autonomy, as discussed above [25]. We suggest that patients can be supported in this process by using PDAs, which can be implemented within the tiered layered staged informed consent model.

## 4. Patient Decision Aids

PDAs are defined as tools developed to help patients make specific, informed choices in health-related decisions that align with their own values [8,35,36]. PDAs have three goals in common: (I) to inform people about the options, risk and benefits of the intervention; (II) to stimulate active participation of the patient in the decision process; and (III) to help people consider their own values and make choices congruent with these values [36]. These goals are clearly in line with the goals of informed consent as discussed above. Decision aids can occur in many forms, varying from an information leaflet to an interactive online tool and are used in both screening and treatment decisions. 

Stimulating patients to consider their own values can be aided by a VCM, which helps patients to identify decisions that are most congruent with their values and preferences [37]. The importance of values clarification in PDAs has been emphasised by the International Patient Decision Aid Standards (IPDAS) Collaboration, who recommends that every PDA should contain a VCM. Given the goals of PDAs, values clarification is inevitable because it is assumed that patients with clear insights of their own values and preferences are more likely to make choices that are aligned with these values and preferences [37]. A VCM can be both implicit (e.g., patient thinks about his options) or explicit (e.g., patient ranks different elements of the decision based on their feeling of importance) [37].

Both implicit and explicit VCMs make use of deliberative processes, which are here defined as conscious and analytical processes [38]. These processes may lead to more value-congruent decisions and a higher level of satisfaction with the choice that people have to make, because deliberative processes align with people’s expectations on how health-related decisions have to be made [38]. Deliberative processes are incorporated in VCMs assuming that people need help with the consideration of their own values and preferences [39]. However, it is not proven that solely applying deliberative processes will result in in better considerations of people’s values and preferences [40]. Deliberative processes might even have a negative influence on people’s natural ability to distinguish between relevant and irrelevant information, which can lead to a higher level of indecisiveness in decision making [38]. Intuitive processes stimulate the ability to separate relevant from irrelevant information. Intuitive processes are here defined as simple decision strategies that rely on less conscious, cognitive processes [38]. Such processes can improve decision making because they allow for the incorporation of feelings and emotions in the decision and the integration of large amount of information [38,41]. However, including feelings and emotions in decision making can also result in decisions that are incongruent with people’s values. Further, choices may be less reproducible for people because they may be influenced by temporary moods or emotions rather than thorough reasoning [38].

Since deliberative and intuitive processes have advantages and disadvantages, it might be preferable to combines these processes. de Vries et al. showed that although it might be difficult to distinguish between deliberative and intuitive processes, they seem to have different effects on decision making in PDAs [38]. 

Combining deliberation and intuition might be specifically interesting in the context of epigenetic risk-stratified screening cancer screening for two reasons. The first reason is related to an important advantage of intuitive processes, which is the implicit integration of information that enables processing large amount of information. This implicit integration can be caused by affective cues or gut feelings and can be of great value in the context of cancer screening because of the large amount of information that will be provided to patients. The second reason is related to a strength of deliberation, which is that deliberation may support patients in expressing their preferences. This is of particular interest in the context of cancer screening because the decision to participate is more preference-sensitive compared to regular treatment decisions [38]. For example, regular treatment choices such as the treatment of an infection with specific types of antibiotics have clear outcomes in terms of benefit and efficiency, and the trade-offs between risks and benefits are limited. However, the outcome of epigenetic cancer screening might be more uncertain and includes trade-offs between risks and benefits, e.g., between quantity and quality of life, and between the benefits and costs of changing lifestyle. The trade-offs that people are willing to make are likely to differ because of personal values and characteristics such as attitude toward uncertainty, values attached to peace of mind, and magnitude of ones fear of cancer [19]. This makes the decision on participating in epigenetic cancer screening more preference sensitive compared to most regular treatment decisions. 

Additionally, cancer screening is usually embedded in nationwide programmes for which people receive a written invitation. In principle, people do not have face-to-face with a medical professional that could help with clarifying people’s values in nationwide screening programmes. Although it might be possible to contact a medical professional, like one’s general practitioner, this is not standard practice. This makes it even more important that patients are capable of clarifying and expressing their preferences independently. Overall, it is vital that the information provided is balanced and neutral, which will limit the possibility of steering. This can be achieved by providing both survival and death rates and by providing absolute risks rather than relative risks [42].

## 5. Framework

**Figure 1:**
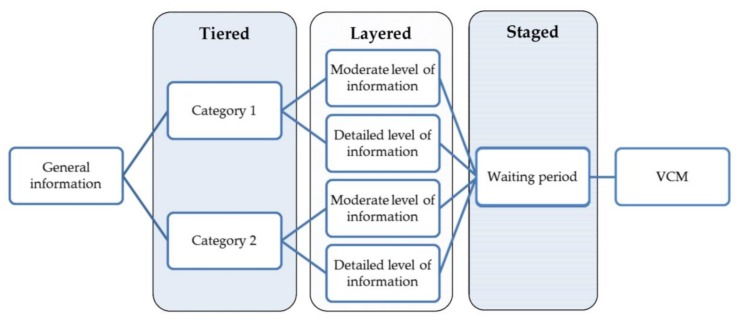
Framework to guide the development of Patient Decision Aids (PDAs) within the tiered layered staged informed consent model. VCM: value clarification methods.

We propose a framework to guide the development of PDAs in the field of epigenetic risk-stratified cancer screening for use within the tiered layered staged informed consent model. The proposed framework incorporates a VCM and a combination of intuitive and deliberative processes (Figure 1). 

The first part of the PDA should consist of a short section with general global information on the screening programme, including the aim of the test, the target population, and the methods that will be used during the test to enable risk prediction. 

After the general information, the PDA should be tiered into different categories. These categories are flexible and can be chosen for each specific screening programme, depending on the outcome of the screening. These categories should be chosen in such a way that they are meaningful to patients, which can be examined by focus groups or surveys. Besides these patient’s preferences, ethical considerations should also be taken into account in discerning relevant categories. Categories can consists of different cancers, but they could also contain several types of unsolicited findings. A specific category, such as unsolicited findings, could then be subdivided into different categories based on condition-specific characteristics like age of onset, action ability or disease severity [32]. 

We want to illustrate these categories the WID test [17]. Implementation of the WID test could mean that the tiered part of the informed consent model consist of three categories. The first category contains the risk predictions for the four cancers, the second category contains unsolicited findings, which could be subdivided into medically actionable unsolicited findings (e.g., lung cancer or diabetes), and unsolicited findings which are not directly medically actionable but could be actionable in terms of procreation, career planning and other life decisions. Subsequently, the information that is presented in a particular category should be layered, as it is expected that the starting knowledge and desired level of knowledge might differ for each individual. Adopting several layers of information with increasing levels of complexity might contribute to a more personalised decision aid that suits to participants starting knowledge. The first layer of information should include all basic information regarding the test, including resulting risk prediction, the expected false positive/negative results, and over-diagnosis. The other layers can contain more in-depth information, which can consist of specific characteristics of cancers, the interplay of genes that are used in screening with epigenetic testing, and information on that the test might reveal about lifestyle and previous exposures.

The next step in the PDA should include a waiting period, possibly with a distraction from the choice people are facing. This waiting period might improve the quality of the decision because it allows people to distance themselves from the decision, which limits the possible influence of strong feelings and emotions that can be evoked during the previous phase of the PDA [37,38]. The optimal length of a waiting period depends on the context in which the PDA is implemented. A distraction of the decision has been shown to improve intuitive processes and may improve decision-making in complex choices.

The waiting period is followed by a VCM. The VCM should be implemented in a later phase of the PDA because this will allow people to consider all relevant information on the decision, instead of solely basing their decision on the aspects of the decision problem that they are familiar with. People can use the information that was provided in the previous phase of the PDA and deliberate on which elements are important to them and suit their values and preferences. The VCM is placed after the waiting period because this will prevent that choices in the VCM are driven by strong emotions [38].

## 6. Conclusions

Given the complex characteristics of new cancer screening programmes that include epigenetic testing and psychological insight that have shown that people have limited rationality and cognitive biases, new strategies are needed to ensure that informed consent reflects an autonomous choice. This processing of information is necessary because solely supplying patients with information insufficiently promotes a choice that is made with understanding and in line with their values. We proposed a framework to guide the development of patient decision aids within the tiered layered staged informed consent model to support informed consent procedures and promote autonomous choices. This framework can be used as guidance, but cannot guarantee that patients actually use the resulting PDA and make autonomous choices. Hence, the responsibility to verify whether a patient is well-informed and the responsibility to obtain informed consent lies with the health care professional or government, depending on the screening context. In addition, more empirical research is needed on how people respond to risk information during medical decision making, and on future strategies to operationalise deliberative and intuitive processes in PDAs
